# Spontaneous Retroperitoneal Hematoma and Membranous Glomerulonephritis; a Case Report

**Published:** 2020-01-25

**Authors:** Foroogh Sabzghabaei, Mohammad Reza Babaei, Asaad Moradi, Behnam Shakiba

**Affiliations:** 1 Department of Internal Medicine, Firoozgar Hospital, Iran University of Medical Science, Tehran, Iran.; 2 Interventional radiology Unit, Firoozgar Hospital, Iran University of Medical Science, Tehran, Iran.; 3 Urology Department, Firoozgar Hospital, Iran University of Medical Science, Tehran, Iran.

**Keywords:** Retroperitoneal space, hematoma, glomerulonephritis, membranous

## Abstract

Spontaneous retroperitoneal hematoma (SRH) is a rare finding which is usually accompanied with anticoagulant and/or antiplatelet aggregation therapy. We describe a patient with a rare presentation of SRH and membranous glomerulonephritis with diffuse visceral arterial micro aneurysms due to medium to small size vasculitis and weakly positive antinuclear antibody (ANA). To the authors’ knowledge, this is a unique report, which does not have any serologic confirmation of specific vasculitis.

## Introduction

Spontaneous Retroperitoneal Hematoma (SRH) is an uncommon clinical entity, which is usually concomitant with anticoagulant or antiplatelet aggregation use (1). Solely, in about 15% of the cases there is no history of these medications, two third of which occur due to vascular causes (1). Hereby, we describe a rare case of SRH with clinical findings of medium to small size vasculitis without any serologic documentation of a specific disease.

## Case presentation:

A 58-year-old Iranian man presented to the emergency department with the chief complaint of generalized abdominal pain lasting for over an hour with radiation to the lumbar region. He had none of the symptoms of nausea, vomiting and diarrhea. In the first visit, he was afebrile with normal vital signs. On physical examination, his abdomen was soft and not distended; however, mild general abdominal tenderness was recognized. He had no history of other illnesses, trauma and drug abuse. His pain disappeared after an hour without any treatment. 

Laboratory test results, which was performed at the time of admission, revealed hemoglobin (Hb)= 9 gr/dl (normal: 12-16), erythrocyte sedimentation rate (ESR)= 102 mm/h (normal: 0-20), serum creatinine (Cr)= 2.5 mg/dl (normal: 0.8-1.3), serum albumin= 2.3 gr/dl (normal :4), serum amylase= 65 U/l (normal<100), lactate dehydrogenase (LDH)= 962 U/ml (normal: 225-500), normal white blood cell count, platelet count, prothrombin and partial thromboplastin time (PT and PTT), and normal aminotransferases level.

In urinalysis, urine sediment pH: 5.5, osmolality: 500 mosmol/kg, protein: 4+, WBC: 3-5/HPF, and RBC: 1-2/ HPF was observed. 24-hour urine protein level was 4.8 gram.

**Figure 1:**
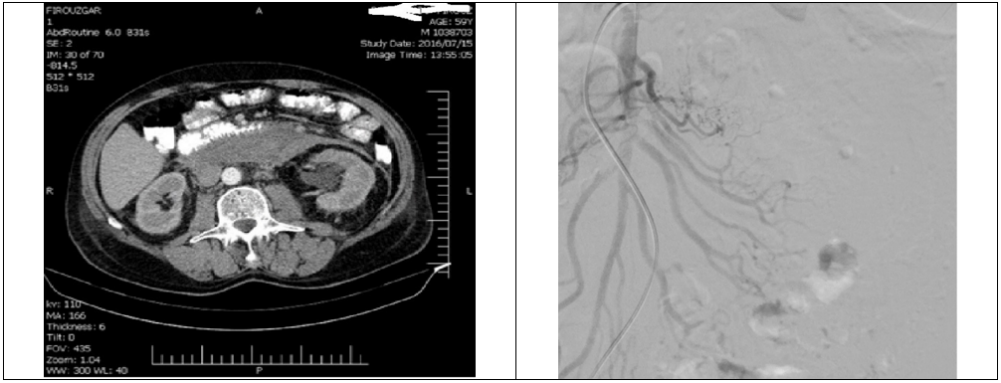
Large retroperitoneal hematoma on the first day of admission without connection to any visceral organ (left image), abdominal angiography with multiple pseudo-aneurysms in multiple splanchnic arteries, superior mesenteric artery and its branches, and renal arteries (right image)

Total abdominal ultrasonography on admission was unremarkable. Upper gastrointestinal endoscopy and colonoscopy only revealed a small gastric ulcer with benign pathology. Abdominopelvic computed tomography (CT) showed large retroperitoneal hematoma without any connection to visceral organs (Figure 1).

Because there was no history of abdominal trauma or anticoagulant use, abdominal angiography was performed, in which presence of multiple visceral and renal small artery aneurysms was reported (Figure 1).

Although there was no hematoma expansion during admission and the patient’s general condition remained good with stable vital signs, his serum Cr increased to 4.8 mg/dl on the 7^th^ day, before contrast medium prescription. The results of an extensive serologic panel for collagen vascular diseases revealed only a positive antinuclear antibody (ANA) =1/160 with speckled pattern. Kidney biopsy was performed and treatment with methyl prednisolone (1gr/d for 3 days) and cyclophosphamide was started with the possible diagnosis of medium to small size vasculitis. Renal pathology was compatible with membranous and focal crescentic glomerulonephritis.

After 8 weeks of treatment his serum Cr and urine protein decreased to 1.7 mg/dl and 1200 mg/24h, respectively. These values were 1.4 mg/dl and 235 mg/24h after 6 months. Abdominal CT angiography was performed after 8 months, which confirmed the disappearance of all visceral micro aneurysms. 

## Discussion:

We have described a patient who presented to emergency department with SRH and membranous glomerulonephritis (MGN) without any laboratory and clinical evidence of any type of vasculitis. SRH is an uncommon disease, which is related to anticoagulant or antiplatelet consumption in 85% of the cases (1). In the remaining 15%, the most frequent causes are renal carcinoma and angiomiolipoma, adrenal lesions (2), and rupture of splanchnic arteries aneurysms due to vasculitis (3, 4).

Multiple aneurysms of splanchnic arteries and renal arteries accompanied with an elevated ESR, low albumin, acute kidney injury and proteinuria without any symptoms or signs of infection strongly define systemic vasculitis, especially involving medium to small size vessels (4). Wegener, Churg- Strauss and microscopic Polyarteritis Nodosa (PAN) are the most frequent diagnoses in this category (5). Absence of pulmonary involvement, eosinophilia, palpable cutaneous purpura, positive anti neutrophil cytoplasmic antibody (ANCA), and presence of MGN beside focal crescents make these diagnoses unlikely. 

The diagnosis of vasculitis is typically made by pathological study, although angiographic findings have also been used frequently (4, 6). A special aspect of our patient was that he had nephrotic range proteinuria and MGN. To our knowledge there is no correlation between PAN and MGN (7, 8) and beside a positive ANA, systemic lupus erythematous (SLE) may be among the differential diagnosis. However, systemic necrotizing vasculitis with aneurysm formation is an uncommon feature of SLE (2-4%) and there are only rare reports of SLE with splanchnic arteries aneurysm rupture, which have been detected through angiography (9, 10).

The patient was treated with immunosuppressive drugs (prednisolone and cyclophosphamide) and a very good response in clinical and para-clinical signs and symptoms was observed after 6 months including Cr=1.4, normal complete blood count and ESR, more than 50 percent decrease in proteinuria and specifically the disappearance of micro aneurysms; yet, we could not differentiate SLE from other systemic vasculitis diseases. Fortunately, their treatment plan is almost similar.

## Conclusion: 

Membranous glomerulonephritis (MGN) without any laboratory and clinical evidence of any type of vasculitis may be a cause of SRH. It should be stated that the present study is a case report and case reports are classified in the low level in the hierarchy of evidence.

## Ethical consideration

The scientific value of presenting this case was fully described to the patient and an informed consent was obtained from him before submission. All procedures performed in the present study were in accordance with the standards of the Ethical Committee of Iran University of Medical Sciences and the 1964 Helsinki Declaration.
